# A memristive circuit for self-organized network topology formation based on guided axon growth

**DOI:** 10.1038/s41598-024-67400-3

**Published:** 2024-07-18

**Authors:** Sebastian Jenderny, Karlheinz Ochs, Daniel Xue

**Affiliations:** 1 Chair of Digital Communication Systems, Ruhr-University Bochum, Universitätsstraße 150, 44801 Bochum, Germany; 2 Department of Electrical and Computer Engineering, University of Virginia, Main Office: Room C210 Thornton Hall, 351 McCormick Road, PO Box 400743, Charlottesville, 22904 USA

**Keywords:** Electrical and electronic engineering, Network models, Network models

## Abstract

Circuit implementations of neuronal networks so far have been focusing on synaptic weight changes as network growth principles. Besides these weight changes, however, it is also useful to incorporate additional network growth principles such as guided axon growth and pruning. These allow for dynamical signal delays and a higher degree of self-organization, and can thus lead to novel circuit design principles. In this work we develop an ideal, bio-inspired electrical circuit mimicking growth and pruning controlled by guidance cues. The circuit is based on memristively coupled neuronal oscillators. As coupling element, we use memsensors consisting of a general sensor, two gradient sensors, and two memristors. The oscillators and memsensors are arranged in a grid structure, where oscillators and memsensors realize nodes and edges, respectively. This allows for arbitrary 2D growth scenarios with axon growth controlled by guidance cues. Simulation results show that the circuit successfully mimics a biological example in which two neurons initially grow towards two target neurons, where undesired connections are pruned later on.

## Introduction

The way a nerve network grows fundamentally affects its functionality. Understanding the underlying principles is not only relevant from a biological point of view, but also of great interest for technical implementations of neuronal networks. Up to now, most technical approaches focus on the growth and change of synapses. Based on biological findings, e.g. learning rules such as spike-timing-dependent plasticity (STDP) have been derived and used for neuromorphic circuits^1,2^. From a biological point of view, however, varying synaptic coupling strengths are only one aspect among many others that shape the structure of a neuronal network. Especially relevant, although often neglected, is axon growth, since it is actually this aspect that forms the connectome. A further aspect of axon growth is the emergence of signal transmission delays. For instance, transmitting an action potential of 5 ms duration across a one-meter-long axon takes 1.6–400 longer than the action potential itself^3,4^. Thus, axons directly influence synaptic learning rules such as STDP, and should not be neglected for neuronal functionality. Considering axon growth principles for technical implementations therefore offers both a functional advantage and a higher degree of self-organization for network growth.

Growth of electrical circuits has for example been considered in the context of a circuit tile assembly model^5–7^. This circuit model, however, does only account for axon growth in a structural sense, as it neglects oscillatory signal (re)generation and propagation. A biologically abstract circuit implementation has been presented in^3^ and is based on memristive Jaumann-structures and delay lines. Here, memristors are nonlinear resistors whose resistance depends on past signals and is maintained when the supplying voltage or current is turned off. A more bio-inspired circuit approach has been proposed in^8^, where neuronal oscillators are coupled via memristors to implement axon growth and pruning.

All these circuit models account for axon growth, but neglect guided growth. How axons are guided during their growth is an active field of research in biology, see e.g.^9–11^. Since this aspect contributes to the self-organizing ability of neuronal networks, considering it for hardware realizations can improve the self-structuring of circuits. Mathematical models for guided axon growth have been discussed in^12,13^. Electrical circuit implementations of this aspect are extremely rare. Recently, an approach making use of memristive sensors in combination with delay lines and Jaumann structures has been reported in^14^. This approach is, however, a very specific implementation of one growth principle and as such not well usable for extending it towards more detailed growth mechanisms such as pruning. Our aim in this work is to design an electrical circuit that can mimic guided axon growth and pruning in a bio-inspired way. Unlike existing circuit implementations of unguided axon growth, this approach allows for controlled growth that affects both the functionality and the structure of the circuit. In contrast to^14^, the bio-inspired nature of our circuit also allows additional growth aspects to be incorporated more easily. To achieve these goals, we use memristively coupled neuronal oscillators. This is inspired by biologically reasonable axon models relying on Hodgkin-Huxley models^15^ coupled via resistors, see for example the McNeal^16,17^ or the spatially extended nonlinear node (SENN) model^17,18^. The use of memristors is especially popular for neuromorphic applications, where memristors are, for instance, utilized as synapses^19,20^. Moreover, they have also been considered for neuron models such as the Hodgkin-Huxley model^21^, the Morris-Lecar model^22^, and the Hindmarsh-Rose model^23,24^. In this work, memristors enable us to achieve a permanent growth of axon paths, similar to^8^.

To verify the proposed electrical circuit, we use a wave digital simulation^25^. The wave digital method has, for example, successfully been applied to simulate both neuronal oscillators^26,27^ and neuronal oscillator networks^8,28^.

The remainder of this manuscript is structured as follows: In Section “Circuit model”, circuit models for neuron building blocks are presented and used to design an overall circuit for guided axon growth. In Section “Simulation results and discussion”, simulation results for an axon growth and pruning example taken from the neocortex of mice are discussed. Concluding remarks are given in Section “Conclusion”.

## Circuit model

### Neuron building blocks

Input signals received by neurons are forwarded to their axons that are long and thin extensions of the main body. The first axon part is called axon hillock and typically initiates the generation of a spiking voltage signal called action potential. This action potential is transmitted via the remaining axon that is typically insulated except for distinct points. The latter are called nodes of Ranvier and exhibit ion channels that regenerate the incoming action potential. In this work, we simply call the parts of an axon that connect nodes of Ranvier axon segments. The electrical signal transmission stops at the end of an axon, where a chemical transmission to the next neuron is carried out via synapses. In the following, we discuss the modeling of these elementary building blocks of a neuron.

#### Axon hillock, node of Ranvier, and axon segment

Axon hillocks and nodes of Ranvier are functionally similar in that they are both capable of generating action potentials. In this work, the only difference between the two is that only axon hillocks receive external input currents *j*. They can in general be modeled by any neuronal oscillator. In this work, we use the equivalent electrical circuit of the Morris-Lecar model, because it is mathematically less complex than e.g. the Hodgkin-Huxley model, but still biologically reasonable, cf.^8^. Since typical neurons contain sodium and potassium channels, we replace the original model’s calcium channels by sodium channels , cf.^8,27,29^. The resulting model equations read

1a $$C\dot{u} = j - G_{{\text{L}}} \left[ {u - E_{{\text{L}}} } \right] - g_{{{\text{Na}}}} (u)\left[ {u - E_{{{\text{Na}}}} } \right] - W_{{\text{K}}} \left[ {u - E_{{\text{K}}} } \right]{\mkern 1mu} ,\quad g_{{{\text{Na}}}} (u) = \frac{{G_{{{\text{Na}}}} }}{2}\left[ {1 + {\text{tanh}}\left( {\frac{{u - U_{{{\text{Na}}1}} }}{{U_{{{\text{Na}}2}} }}} \right)} \right]{\mkern 1mu} ,\quad W_{{\text{K}}} = z_{{\text{K}}} G_{{\text{K}}}$$

1b $$\begin{aligned} {\dot{z}}_{\textrm{K}}&= \left[ f(u) -z_{\textrm{K}} \right] F_{\textrm{K}}\textrm{cosh}\left( \frac{u-U_{\textrm{K}1}}{2U_{\textrm{K}2}}\right) \,,&f(u)&= \frac{1}{2}\left[ 1+ \textrm{tanh}\left( \frac{u-U_{\textrm{K}1}}{U_{\textrm{K}2}}\right) \right] \,. \end{aligned}$$

Here, *u* is the membrane potential. The sodium channels are described by the conductance function $g_{{{\text{Na}}}} (u)$, and the potassium channels are characterized by the memristance $W_{{\text{K}}}$ governed by the fraction of open channels $z_{{\text{K}}}$. The voltage-dependent opening and closing of these channels is described by *f*(*u*). Here, $f(u) = 0$ indicates that all channels of the considered ion type are closed, $0< f(u) < 1$ means that some of the channels are open, and $f(u)= 1$ corresponds to all channels being open. The combined channel equations govern a threshold-based oscillation behavior: When the input current *j* is large enough to increase *u* above a certain threshold voltage, $g_{{{\text{Na}}}}$ increases and triggers a rapid and strong rise of *u* due to the positive resting potential $E_{{{\text{Na}}}}$. This leads to positive voltage values that result in an increase of $W_{{\text{K}}}$ and an activation of $E_{{\text{K}}}$. Since $E_{{\text{K}}}$ is negative, *u* drops back to the resting potential that is close to $E_{{\text{L}}}$. Parameters utilized in this work are shown in Table 1 and are based on^29^. The corresponding equivalent electrical circuit is depicted in Fig. 3b. Note that we only apply an external current *j* to axon hillock models that are to grow from the beginning.

**Table 1:** Parameters for node of Ranvier and axon hillock.

Parameter	Variable	Value
Capacitance	*C*	2 $\textrm{nF}$
Leakage conductance	$G_\mathrm{L}$	20 $\mu \textrm{S}$
Leakage resting potential	$E_\mathrm{L}$	− 70 $\textrm{mV}$
Sodium conductance	$G_\mathrm{Na}$	0.2 $\textrm{mS}$
Sodium resting potential	$E_\mathrm{Na}$	50 $\textrm{mV}$
Sodium threshold voltage	$U_{\textrm{Na1}}$	− 1.2 $\textrm{mV}$
Sodium edge steepness	$U_{\textrm{Na2}}$	18 $\textrm{mV}$
Potassium conductance	$G_\mathrm{K}$	0.2 $\textrm{mS}$
Potassium resting potential	$E_\mathrm{K}$	$-90$ $\textrm{mV}$
Potassium threshold voltage	$U_{\textrm{K1}}$	0 $\textrm{mV}$
Potassium edge steepness	$U_{\textrm{K2}}$	10 $\textrm{mV}$
Potassium channel opening rate	$F_\mathrm{K}$	66.7 $\textrm{Hz}$

To model an axon segment, one can use a constant resistor that interconnects the Morris-Lecar circuits in series^8^. We call the alternating interconnection of Morris-Lecar circuits and resistors a static axon model, because it cannot change its structure, but exhibits a signal transmission delay. The latter stems from the fact that subsequent oscillators can only begin their oscillation once previous ones started, leading to a delay increasing with each oscillator added.

#### Synapse

Synapses form when an axon contacts the dendrites of another neuron. Synaptic transmission occurs when an action potential reaches the end of an axon, leading to the opening of ion channels at the dendrites of the postsynaptic neuron. As this work focuses on axon guidance, we use a synapse model mimicking the channel opening and neglect short- and longterm memory aspects typically associated with synapses^30^. In particular, we deploy a memristor model comparable to the potassium channel memristor of the Morris-Lecar model. Similar to an axon segment model, the synapse model is connected in series with a node of Ranvier and an axon hillock, see Fig. 3. The model equations read

2 $$W_{{\text{s}}} (z_{{\text{s}}} ) = G_{{{\text{s1}}}} z_{{\text{s}}} + G_{{{\text{s0}}}} ,\quad \dot{z}_{{\text{s}}} = \left[ {\frac{1}{2}\left[ {1 + {\text{tanh}}\left( {\frac{{u - U_{{{\text{s}}1}} }}{{U_{{{\text{s}}2}} }}} \right)} \right] - z_{{\text{s}}} } \right]F_{{\text{s}}} ,$$

where $W_\mathrm{s}$ is the synaptic memductance and $z_\mathrm{s}$ is its state variable. $G_\mathrm{s1}=20$ μS is the maximum conductance, $G_\mathrm{s1}=5$ μS is the minimum conductance, $U_\mathrm{s1}=40\,\textrm{mV}$ is the threshold voltage, $U_\mathrm{s2}=1\,\textrm{mV}$ is the edge steepness, and $F_\mathrm{s}=3\,\textrm{kHz}$ denotes the opening rate of synaptic channels. Manufactured memristors with similar resistance ranges are e.g. reported on in^31,32^.

### Growth concept

**Figure 1:**
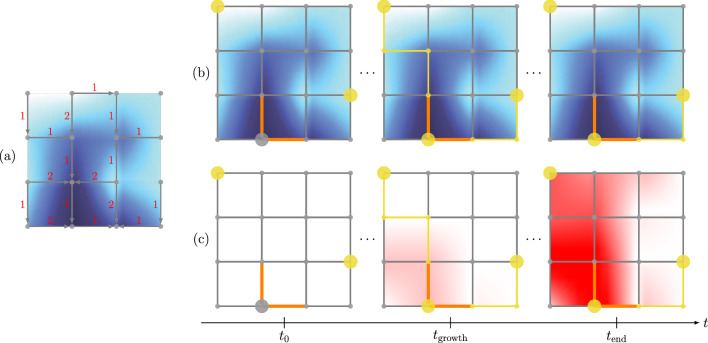
Illustration of the growth concept and pruning concept. Here, large circles represent axon hillocks, where only the yellow ones are active. Small circles are potential nodes of Ranvier, with the yellow ones denoting grown nodes of Ranvier. Thin lines are potential axon segments, where the yellow ones denote the actually grown ones. Thick orange lines are potential synapses that are formed when connected to a grown axon segment. (**a**) Directional derivatives of the growth map are highlighted in red at the edges of the grid. (**b**) Growth map with grid structure due to gradient-based network growth. (**c**) Pruning map with the same grid structure as in (**b**). Note that $t_0$ is the start time, $t_\mathrm{growth}$ is the time at which growth is completed, and $t_\mathrm{end}$ is the time at which pruning is completed.

In biology, the growth of axons is guided by guidance cues, where axons follow a path along concentration gradients^33^. We denote these growth-controlling cues as growth cues. The aim of this work is to implement this growth behavior in a bio-inspired way by an electrical circuit. For this purpose, we assume a regular grid structure as the environment in which growth can occur, see Fig. 1b.

Each grid point is either a potential node of Ranvier (small circles) or an axon hillock (large circles), where the latter are only placed at start and target positions. Edges between potential nodes of Ranvier are potential axon segments (thin lines), and edges between axon hillocks and potential nodes of Ranvier are either potential axon segments or potential synapses (thick orange lines). The positions of the targets and synapses need to be determined in advance. If the sensed directional derivatives of the concentration map, see Fig. 1a, are larger than a certain threshold, the axon segments grow towards the next node of Ranvier. This is illustrated in Fig. 1b for a threshold of 0. Here, active axon hillocks as well as grown nodes of Ranvier and axon segments are highlighted in yellow.

### Pruning concept

While we associate the addition of new axon segments with growth, we refer to the removal of axon segments as pruning. According to^34,35^, one often distinguishes between small-scale and large-scale axonal pruning. The former describes the reduction of the number of interconnections between two neurons, while large-scale pruning is associated with removing the entire link between two neurons. This type of pruning is assumed to be predetermined and occurs e.g. for removing unnecessary or false interconnections. In this work, we focus on the functional connection structure of neurons, i.e., whether a connection exists or not. Since small scale pruning has no influence on this, we only consider large scale pruning.

As a simple modeling approach, we assume that pruning cues are generated at the target neuron only when it is connected to the outgrowing neuron. This is illustrated in Fig. 1b. This approach is inspired by an axon pruning example observed from the neocortex of mice, see^34^. Note that different neurons can be sensitive to different pruning cues, see e.g.^34^. For reasons of simplicity, however, we account for a single pruning cue that can prune axons of different neurons.

### Growth- and pruning-cue sensitive axon segment

Based on^8^, axon growth in the proposed grid structure can be interpreted as the length increase of an axon segment until a conductive path between two nodes of Ranviers emerges. In a similar sense, pruning is then associated with the length decrease of an axon segment. The growth of axon segments should depend on the sensed directional derivatives of the growth concentration and should only occur at consecutive positions. The latter can be ensured by transmitting a control signal to the current ending position(s) of the axon. To allow for pruning, the axon segment should also be able to sense the pruning cue concentration.

From an electrical circuit point of view, the length increase or decrease of an axon segment is equivalent to a decreasing or increasing resistance, respectively. An electrical circuit model for the axon segment is hence required to dynamically adjust its resistance value. This adjustment should depend on (i) a control signal triggering the growth, (ii) external stimuli representing directional derivatives of a growth cue concentration, and (iii) external stimuli that account for a pruning cue concentration. For these reasons, we use a memristor in combination with multiple sensors, which we call memsensor, cf.^36^. Note that memristors acting as sensors themselves have also been reported, see^37^. We neglect this option in this work, since we believe that with the current state of art, memristors in combination with sensors offer a better implementable and flexible approach due to less limitations of available devices.

**Figure 2:**
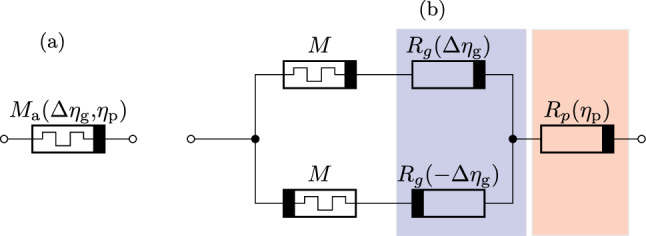
(**a**) Memsensor as axon segment model and (**b**) its equivalent circuit consisting of memristors, gradient sensors (blue), and a general sensor (red).

The memsensor model considered in this work is shown in Fig. 2 and consists of two anti-parallel memristors, two anti-parallel gradient sensors, and one general sensor. Note that the anti-parallel interconnection of memristors and gradient sensors enables a growth in both directions. Moreover, memristors account for a non-volatile growth of axon segments. The memristor model used in this work yields

3a $$M(z_{{\text{a}}} ) = M_{1} + z_{{\text{a}}} \left[ {M_{0} - M_{1} } \right]{\mkern 1mu} ,\quad \dot{z}_{a} = w(z_{a} )\left[ {S\left[ {\sigma \left( {u - U_{ + } } \right) - \sigma \left( { - u - U_{ - } } \right)} \right] - S_{{\text{r}}} } \right],$$

with the memristance *M*, the memristor state $z_\mathrm{a}$, the heaviside function $\sigma (\cdot )$, and a window function $w(z_\mathrm{a})$ ensuring that $z_\mathrm{a}\in [0,1]$. $S_\mathrm{r}$ denotes the slope of a retention characteristic, which enables an increase of the memristance and thus implements the length decrease due to pruning. The gradient sensor is described by

3b $$\begin{aligned} R_\mathrm{g}(\Delta \eta _\mathrm{g})&= R_{\textrm{g},0} + R_\mathrm{g,1}\left[ 1 - \textrm{tanh}\left( \frac{\Delta \eta _\mathrm{g}-\Delta \eta _\mathrm{g1}}{\Delta \eta _\mathrm{g2}}\right) \right] , \end{aligned}$$

where $\Delta \eta _\mathrm{g}$ is a normalized, dimensionless gradient sensor signal representing the directional derivative of the sensed growth cue concentration $\eta _\mathrm{g}$. The general sensor model reads

3c $$\begin{aligned} R_\mathrm{p}(\eta _\mathrm{p})&= R_\mathrm{p,0} + R_\mathrm{p,1}\left[ 1 + \textrm{tanh}\left( \frac{\eta _\mathrm{p}-\eta _\mathrm{p1}}{\eta _\mathrm{p2}}\right) \right] \,. \end{aligned}$$

Here, $\eta _\mathrm{p}$ is a normalized, dimensionless sensor signal that we associate with the pruning cue concentration. For a technical implementation, the external stimuli representing growth and pruning cue concentrations can be given by e.g. light sources with two different wave lengths. In this sense, two light sensors for two different wave lengths are required.

Under ideal conditions, the functionality of the complete memsensor can be characterized as follows: To disable signal transmission between two oscillators, memristors are initially in their high resistance states $M_1$. In the absence of strong growth cue gradients, gradient sensors are also high ohmic. Choosing $R_{\textrm{p},1}>M_{1}$, the majority of the voltage is present at the sensor. This prevents memristors from switching to $M_1$ and thus signals being transmitted when no growth indication is given. Growth starts when three conditions are met: (i) One of the oscillators connected to the memsensor must be active. (ii) Growth cue gradients are sensed by one of the sensors $R_\mathrm{g}$ and have to exceed a certain threshold value. (iii) The growth cue gradient has to be directed towards the inactive oscillator. If these conditions are met, one memristor switches towards $M_1$, because $R_\mathrm{g}$ of the same direction is now decreased, enabling the memristor voltage to exceed its threshold. This way, the total resistance of the memsensor becomes $M_0+R_{\textrm{g},0}+R_{\textrm{p},0}$. Given that $M_0+R_{\textrm{g},0}+R_{\textrm{p},0}$ is below the minimum resistance that prevents signal transmission, the previously inactive oscillator begins to oscillate. For the chosen Morris-Lecar oscillators, this resistance threshold is roughly $100\,\mathrm {k\Omega }$. Pruning takes place when pruning cues are sensed, leading to an increased $R_p=R_{\textrm{p},1}$. Setting $R_{\textrm{p},1}>M_1$, the complete voltage is now present at the pruning sensor. This way, the memristor with $M_0$ is driven back to its high resistance state $M_1$ via its retention characteristic. The parameters utilized for the memsensor are shown in Table 2. Memristors observed in experimental works with comparable parameters, especially in terms of resistances, can for instance be found in^38–40^. Similar threshold voltages can be achieved when rescaling the oscillator circuit parameters.

**Table 2:** Memsensor parameters.

Parameter	Variable	Value
Low memristance state	$M_0$	10 $\mathrm {k\Omega }$
High memristance state	$M_1$	1 $\mathrm {M\Omega }$
Slope	*S*	4 $\textrm{kHz}$
Slope of retention characteristic	$S_\mathrm{r}$	0.2 $\textrm{Hz}$
Positive threshold voltage	$U_+$	30 $\textrm{mV}$
Negative threshold voltage	$U_-$	− 0.3 $\textrm{V}$
Minimum resistance of gradient sensor	$R_\mathrm{g,0}$	5 $\mathrm {k\Omega }$
Maximum resistance of gradient sensor	$R_\mathrm{g,1}$	1.5 $\mathrm {M\Omega }$
Threshold value for gradient sensor	$\Delta \eta _1$	1
Edge steepness for gradient sensor	$\Delta \eta _2$	1
Minimum resistance of pruning cue sensor	$R_\mathrm{p,0}$	5 $\mathrm {k\Omega }$
Maximum resistance of pruning cue sensor	$R_\mathrm{p,1}$	125 $\mathrm {k\Omega }$
Threshold value for pruning cue sensor	$\eta _1$	3.5
Edge steepness for pruning cue sensor	$\eta _2$	1

### Pruning-cue generation circuit

In order to get closer to biology with our circuit model, we incorporate a dynamic pruning cue generation. For modeling purposes, we assume that neurons only act on local information, i.e. if a synaptic connection at their dendrites exist, or if action potentials are generated at their axon hillock. Based on this, we assume that pruning cues are only generated at the target neuron once they become active, since this is a signal-based indication that a connection to at least one of the outgrowing neurons has been established. Since we, for reasons of simplicity, consider only a single pruning cue that should prune only undesired connections, a directional emission of the cues is also necessary. A model for the pruning cue generation yields

4a $$\begin{aligned} {\dot{\eta }}_{\textrm{p},0}&= \alpha \sigma (u-u_\mathrm{th})-\beta \eta _{\textrm{p},0}\,,{} & {} {}{} & {} \end{aligned}$$

where $\eta _{\textrm{p},0}$ is the pruning cue concentration generated at the axon hillock of a target neuron located at $(x_0,y_0)$ and *u* is the membrane potential of this axon hillock. $\alpha =120$ is the pruning cue generation rate, $\beta =3$ is the pruning cue decay rate, and $u_\mathrm{th}=15\,\textrm{mV}$ is the threshold voltage for generating pruning cues. The damping of the pruning cue emission is described via

4b $$\begin{aligned} \eta _\mathrm{p}&= \frac{1}{2}\left[ \frac{{\textbf{s}}^T{\textbf{r}}}{|{\textbf{s}}||{\textbf{r}}|^2} + 1\right] \frac{\eta _{\textrm{p},0}}{1 + |{\textbf{r}}|}\,,&{\textbf{s}}&= \begin{pmatrix} x_\mathrm{s}-x_0\\ y_\mathrm{s}-y_0 \end{pmatrix}\,,&{\textbf{r}}&= \begin{pmatrix} x-x_0\\ y-y_0 \end{pmatrix}. \end{aligned}$$

Here, (*x*, *y*) is the observed position on the grid, $(x_\mathrm{s},y_\mathrm{s})$ is the position of the synapse whose connected axon should be pruned. The vectors ${\textbf{s}}$ and ${\textbf{r}}$ indicate the directions from target axon hillock to the synapse and observed grid point, respectively, as well as the corresponding distances.

**Figure 3:**
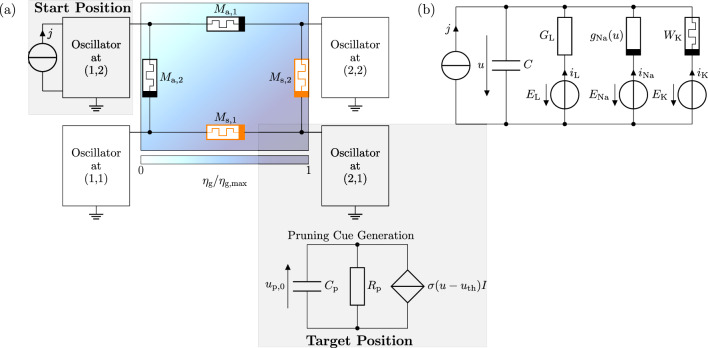
Circuit setup for guided axon growth. (**a**) Complete circuit structure for the minimal example, with synapse memristors in orange and the growth cue concentration as background. (**b**) Morris-Lecar circuit as oscillator model for axon hillocks and nodes of Ranvier.

In a second step, we represent the differential equation (4a) responsible for the cue generation with an equivalent electrical circuit shown in Fig. 3a. Redefining $\alpha = \alpha _1\alpha _2$, with $\alpha _1=3 \cdot 10^3$ and $\alpha _2=40\cdot 10^{-3}$, the circuit is governed by

5 $$\begin{aligned} C_\mathrm{p}{\dot{u}}_{\textrm{p},0}&= \sigma (u-u_\mathrm{th})I - \frac{1}{R_\mathrm{p}}u_{0}\,,&u_{\textrm{p},0}&= \eta _{\textrm{p},0}U_0\,,&R_\mathrm{p}&= \frac{\alpha _1}{\beta }R_0\,,&C_\mathrm{p}&= \frac{1}{\alpha _1}C_0\,,&I&=\alpha _2 I_0\,. \end{aligned}$$

Here, $C_\mathrm{p}$ and $R_\mathrm{p}$ are the capacitance and the resistance of the RC circuit, $u_{\textrm{p},0}$ is the voltage representing the pruning cue concentration at the point of cue generation $(x_0,y_0)$, and *I* is the input current amplitude. $I_0 = 1\,\textrm{A},\, U_0 = 1\,\textrm{V},\, R_0=1\,\mathrm {\Omega }$, and $C_0=1\,\textrm{F}$ are a normalization current, voltage, resistance, and capacitance. With regard to a technical implementation based on light sensors, the RC circuit could be replaced by a light-emitting diode. Note that in our model, the spread of the cue concentration is still mathematically calculated via Eq. (4b).

## Simulation results and discussion

### Minimal example

To demonstrate the general functionality of the circuit concept, let us consider the minimal example shown in Fig. 3a that displays the growth cue concentration as background for the circuit setup. The corresponding circuit consists of four oscillators, one for each of the four possible locations on the $2\times 2$ grid. Oscillators are connected to each other by two memsensors and two synapse memristors highlighted in black and orange, respectively. In the example, only the oscillator related to the position (1, 2) where growth starts is supplied with an external current signal *j*. The latter consists of rectangular shaped pulses, with an amplitude of $2.4$ μA, pulse width $T_\mathrm{pw} = 2\,\textrm{ms}$, and a pause between the pulses of $T_\mathrm{p} = 4\,\textrm{ms}$. The oscillator of the target position at (2, 1) is connected to a pruning cue generation circuit that should dissolve the oscillatory connection via the Memsensor $M_{\textrm{a},1}$. Both oscillators at start and target position are considered as axon hillocks, since they act as the first segment of a neuron.

**Figure 4:**
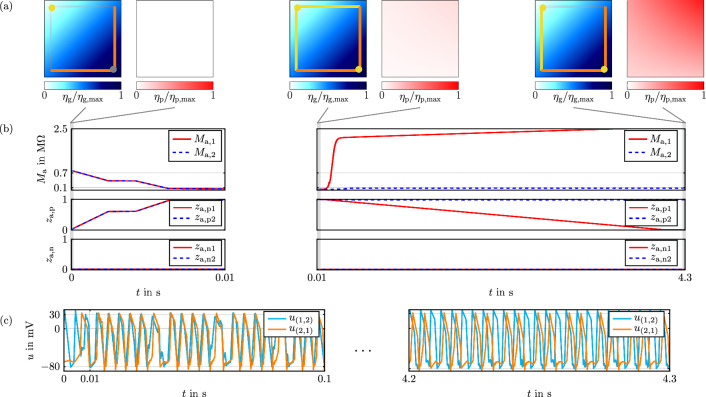
Simulation results for the minimal example. (**a**) The resulting network structure and the growth cue concentration at a specific time point are shown on the left side, with grown axon segments and active axon hillocks marked in yellow, and synaptic connections in orange. The right side contains the pruning cue concentration. (**b**) Total memristances of the memsensor (top), memristor states for positive direction (center), and memristor states for negative direction (bottom). (**c**) Axon hillock voltages.

Results for the setup are depicted in Fig. 4 and are obtained through wave digital simulations. Note that we set $\eta _2=0.5$ to narrow the sensor range in this minimal example. As can be seen from the top of Fig. 4b, both total memristances $M_{\textrm{a},1}$ and $M_{\textrm{a},2}$ decrease below $100\,\mathrm {k\Omega }$ within less than $10\,\textrm{ms}$. This is due the memristors in positive direction switching to the low resistance states, while memristors in negative direction remain in the high resistance states. This can be inferred from the center and bottom of Fig. 4b. $100\,\mathrm {k\Omega }$ is set as threshold value for determining grown connections, since this value permits signal transmission. We have highlighted the corresponding network structure in Fig. 4a for specific time points. The left side shows the growth cue concentration and the network structure, while the right side displays the pruning cue concentration. This shows that shortly after $10\,\textrm{ms}$, at which both connections from position (1,2) to position (2,1) are established, pruning cues are mainly generated in the direction of $M_{\textrm{a},1}$. $R_{\textrm{p},1}$ senses this generated pruning cue concentration and increases, due to which $M_{\textrm{a},1}$ rises as well. This in turn reduces the voltage at the memristor being in the low resistance state, making it slowly switch back to its high resistance state, cf. Fig. 4b. The sharp increase of $M_{\textrm{a},1}$ in the beginning is mainly due to the sensor resistor $R_{\textrm{p},1}$, since the pruning cue concentration is slowly building up. After the concentration and hence the sensor resistance reach saturation, the weaker increase of $M_{\textrm{a},1}$ stems from the memristor that switches back. The connection via $M_{\textrm{a},1}$ is completely dissolved after $4.3\,\textrm{s}$, while the desired connection via $M_{\textrm{a},2}$ is still below $100\,\mathrm {k\Omega }$. This enables a working signal transmission from position (1,2) to position (2,1), as can be seen from Fig. 4c displaying the corresponding oscillator voltages. The example thus shows the successful growth and subsequent pruning of desired and undesired connections.

### Biological example

**Figure 5:**
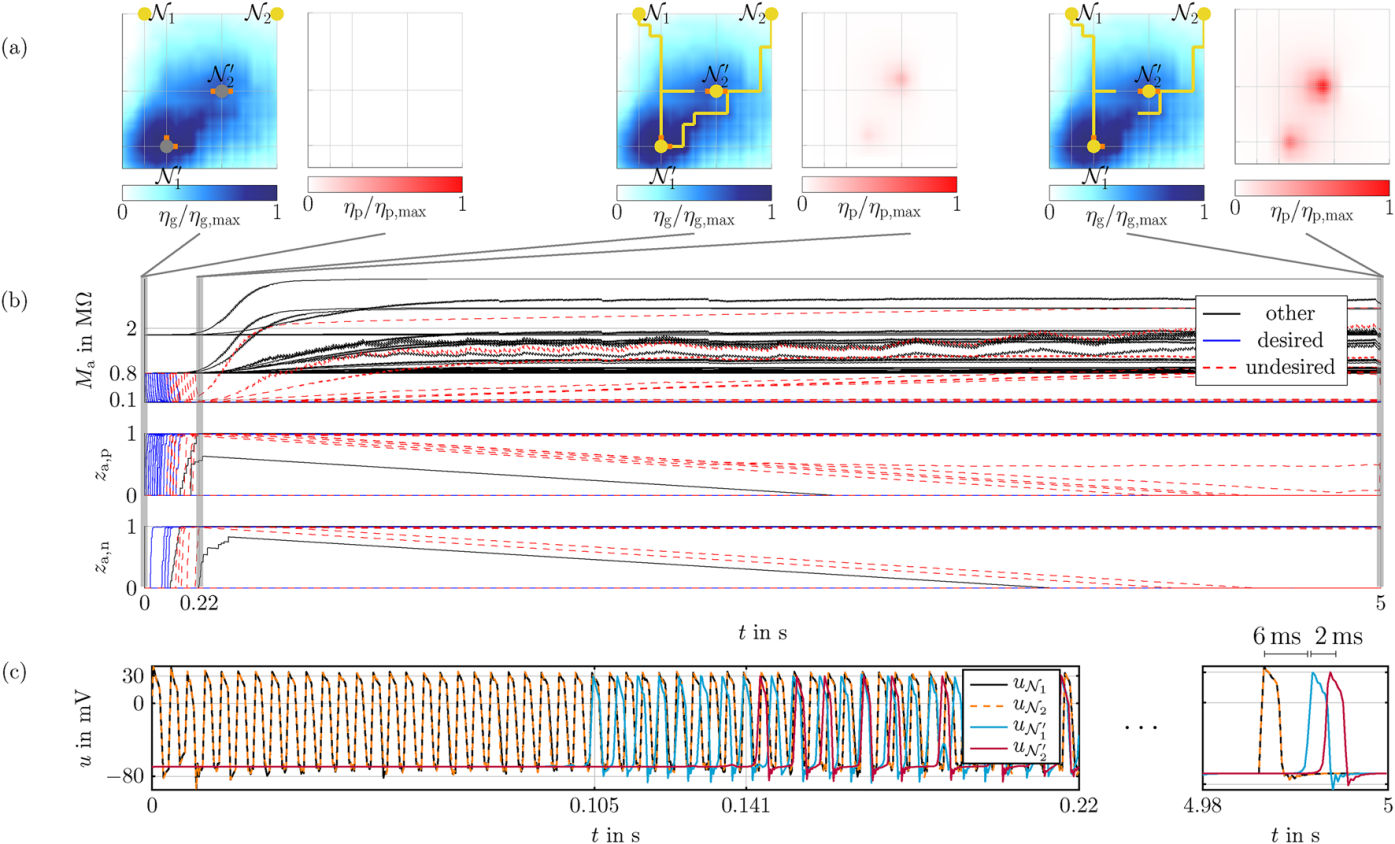
Simulation results for the biologically inspired example. (**a**) The resulting network structure and the growth cue concentration at a specific time point are shown on the left side, while the right side displays the pruning cue concentration. (**b**) Total memristances of the memsensor (top), memristor states for positive direction (center), and memristor states for negative direction (bottom). (**c**) Axon hillock voltages.

In the following, we consider a circuit setup inspired by axon growth and pruning observed from the neocortex of mice^34^. In particular, axons from both a visual cortex neuron ${\mathcal {N}}_1$ and a motor cortex neuron ${\mathcal {N}}_2$ grow towards a spinal cord neuron ${\mathcal {N}}_2'$ and a superior colliculus neuron ${\mathcal {N}}_1'$. Note that the superior colliculus is a visual computation center. However, only the connections from the visual cortex to the superior colliculus and from the motor cortex to the spinal cord are desired. As a result, the false connections between neurons ${\mathcal {N}}_1$ and ${\mathcal {N}}_2'$ and neurons ${\mathcal {N}}_2$ and ${\mathcal {N}}_1'$ are pruned afterwards. As an environment for the growth and pruning, we consider a $15\times 15$ grid, containing 225 Morris-Lecar circuits, 416 memsensors, four synapse memristors, and two RC circuits for generating pruning cues. In this grid, see Fig. 5a, we place the axon hillocks of the growing neurons ${\mathcal {N}}_{1}$ and ${\mathcal {N}}_{2}$ at (5, 15) and (15, 15), respectively. Corresponding Morris-Lecar circuits are excited by the external current signal *j* that is chosen equal to the minimal example. The axon hillocks of the target neurons ${\mathcal {N}}_{1'}$ and ${\mathcal {N}}_{2'}$ are located at (5, 3) and (10, 8), respectively. Synapses are placed above and right of ${\mathcal {N}}_{1'}$, as well as left and right of ${\mathcal {N}}_{2'}$.

Simulation results are shown in Fig. 5. Similar to the minimal example, Fig. 5b displays the total memristances $M_\mathrm{a}$ and both internal memristor states $z_\mathrm{a,p}$ and $z_\mathrm{a,n}$ for the positive and negative direction in the top row, center row, and bottom row, respectively. Blue curves indicate desired connections, red curves indicate connections that should later on be pruned, and black curves are connections that are not part of the directs paths between start and target neurons. Memsensors that sense a sufficiently large growth cue gradient start at memristance values of $800\,\mathrm {k\Omega }$, while all other memsensors start at $1.8\,\mathrm {M\Omega }$. Of the memsensors sensing a large enough growth cue gradient, those connected by a continuous pathway to the oscillators modeling active axon hillocks switch to the low resistance state. This can be inferred from the internal memristor states and indicates that corresponding connections have grown. Overall growth is finished after $\approx 0.22\,\textrm{s}$. Pruning starts earlier, because connections from the start neurons to the target neurons ${\mathcal {N}}_{1'}$ and ${\mathcal {N}}_{2'}$ are established after $0.105\,\textrm{ms}$ and $0.141\,\textrm{ms}$, respectively. These time points can be taken from the axon hillock voltages illustrated in Fig. 5c that shows when the target neurons become active.

As the pruning cue concentration rises, corresponding sensor resistances increase, resulting in rising total memristances $M_\mathrm{a}$ of those connections to be pruned. As in the minimal example, this reduces the voltage of the internal memristors, such that they slowly drop back to the high resistance state. As can be seen from the center and bottom row of Fig. 5b, this high resistance state is reached after $~4.5\,\textrm{s}$ for almost all pruned connections. This demonstrates that self-organized growth and pruning is also possible for larger setups. To highlight the delayed signal transmission that comes with this growth and pruning process, we have excited the target neurons with a separate current pulse at $4.99\,\textrm{s}$, following a short pause. This can be seen from the right side of Fig. 5c and illustrates that signal transmission from ${\mathcal {N}}_{1}$ to ${\mathcal {N}}_{1'}$ and ${\mathcal {N}}_{2}$ to ${\mathcal {N}}_{2}'$ takes $8\,\textrm{ms}$ and $6\,\textrm{ms}$, respectively.

The biologically inspired example also highlights the advantages of the proposed circuit approach compared to the existing one for guided axon growth^14^. First, our approach provides a pruning concept that is missing in the previous work. This pruning improves the self-organized growth of connections and allows for dynamically reconfigurable setups. Second, we deploy a more elaborated memsensor model based on separated memristors and sensors instead of a memristor with sensing abilities. Since the latter are advantageous in functionality, but hardly available as they are very idealized, this makes our approach more flexible for possible implementations. Third, the concept of^14^ relies on circulators that relay voltages from ones axon segment to another. This limits its applicability to branching axons, since each branch halves the transmitted voltage. In contrast to this, our concept makes use of coupled neuronal oscillators that are able to regenerate the transmitted voltage. This way, our concept is well suited for branching axons, as has been demonstrated.

#### Robustness against parameter variations

**Figure 6:**
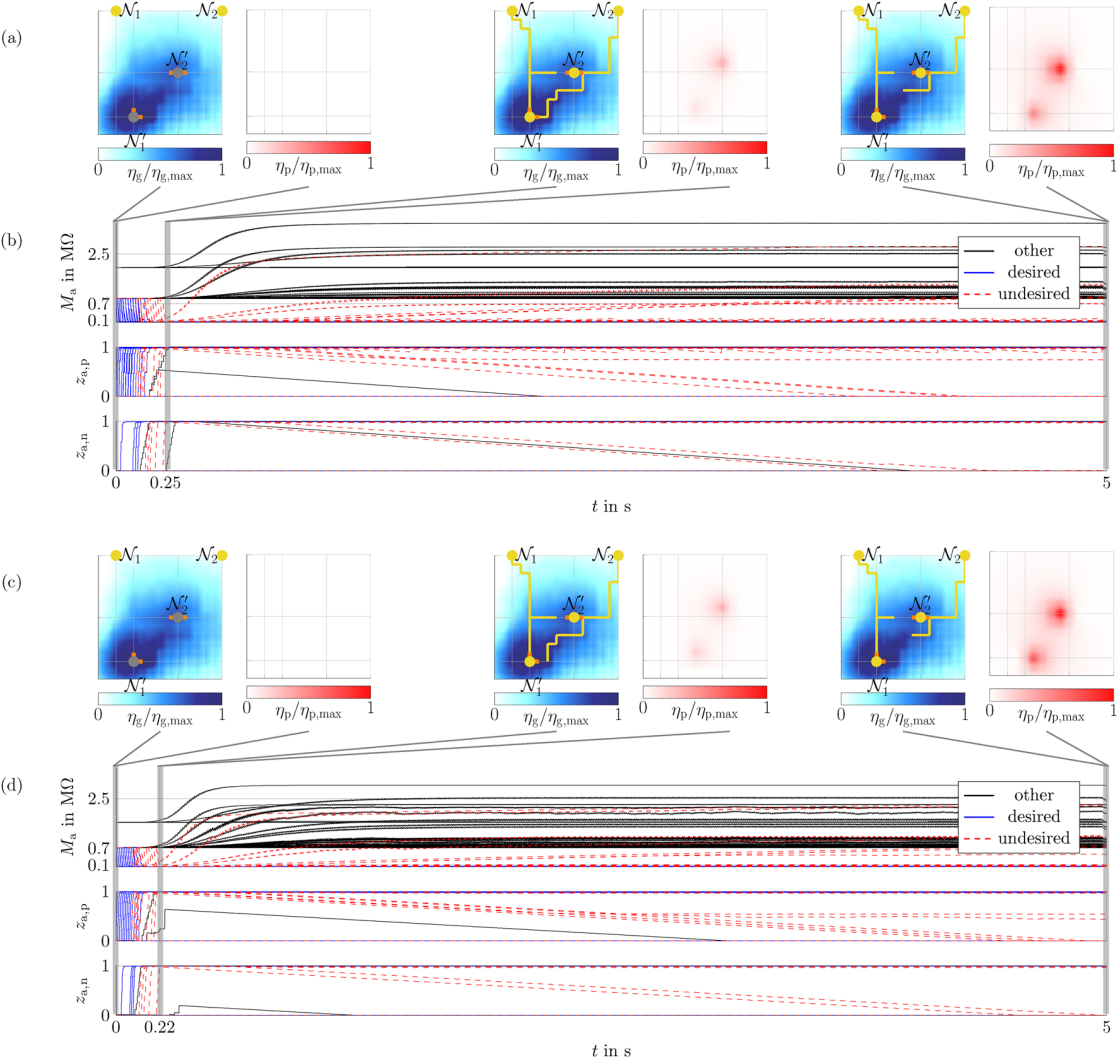
Simulation results for the biologically inspired example with memsensor parameters varied by ± 10%. Results for parameters with $+\,10\%$ deviation are shown in (**a**), (**b**), while (**c**), (**d**) display results for parameters with − 10% deviation. Resulting network structures, growth cue concentrations, and pruning cue concentrations are shown in (**a**), (**c**). Total memristances and internal state variables for positive and negative direction are illustrated in (**b**), (**d**).

To observe the influence of parameters variations on the biologically inspired example, we have investigated two worst-case scenarios where the memsensor parameters are varied by either + 10% or − 10%. Corresponding results are shown in Fig. 6a, b (+ 10%) and Fig. 6c, d (− 10%). As can be seen, the emerging structures with respect to the threshold memristance $100\,\mathrm {k\Omega }$ remain equal except for one element less to be pruned in the case of $+10\%$. Little effect can also be observed for a full pruning of memristors, meaning they returned to their high resistance state. This can again be inferred from the state variables $z_\mathrm{a,p}$ and $z_\mathrm{a,n}$. These state variables show that compared to 0% parameter variation, see Fig. 5, a similar amount of memristors returns to the high resistance state. Differences in the results are due to variations of pruning sensor sensitivity, high resistance states of pruning sensor and memristors, and the threshold voltage of the memristors, as these parameters mainly determine which and how effectively connections are pruned.

## Conclusion

In this work, we have proposed a bio-inspired electrical circuit mimicking axon growth controlled by growth and pruning cues. Our circuit is based on a Morris-Lecar circuit as a node of Ranvier or axon hillock, and a memsensor as axon segment. The memsensor consists of one general sensor, two gradient sensors, and two memristors. The latter enable a permanent growth of axon segments when decreasing their resistance based on an STDP-like mechanism. As an overall and very general architecture, we have used a grid structure, where each node is implemented by the Morris-Lecar circuit and each edge by the memsensor. This way, once the target points are fixed, the setup allows for arbitrary growth paths, because starting points are chosen by just applying a current stimulus to the corresponding Morris-Lecar circuits. We like to stress that the STDP-like mechanism of the growth concept makes it possible to use other neuronal oscillators as well. Note that this requires e.g. the memsensor parameters or the input current signal to be redesigned.

As a bio-inspired application example, we have considered two neurons both growing towards to two target neurons, although two of the resulting connections should be pruned later on. Simulations have verified that our circuit can mimic this behavior in terms of dynamically growing structures that affect the delay of the signal transmission. This underlines the bio-inspired and functional character of the self-organized network topology formation implemented by our circuit.

## Methods

### Wave digital simulation

Wave digital simulations are based on a concept^25^ that provides a run-time efficient algorithm and has a direct correspondence to the reference circuit. Such an algorithm can be derived by port-wisely decomposing the reference circuit and, in a second step, translating each one- and multi-port and their interconnection structures into the wave digital domain using the bijective transformation

6 $$\begin{aligned} a&= u+Ri\,,&b&= u-Ri\,.\end{aligned}$$

Here, *a* is the incoming wave and *b* is the reflected wave. *R* is the port resistance that can be chosen freely as long as $R>0$ holds. An appropriate choice of *R* is, however, important, as it significantly reduces the algorithm’s complexity. For instance, choosing the port resistance of a capacitor with capacitance *C* to $R_C = \frac{T}{2C}$ leads to a delay element in the wave digital domain. Likewise, a resistor with resistance $R_0$ translates to $b=0$ and a resistive voltage source with resistance $R_0$ and voltage *e* leads to $b=e$ when choosing $R=R_0$. For more general information on the wave digital concept, the interested reader is referred to^25^, while wave digital models related to the context of this work can be found in^8,26,27^.

## Data Availability

The data that support the findings of this study are available on request from the corresponding author.
